# Parent-Focused Childhood and Adolescent Overweight and Obesity eHealth Interventions: A Systematic Review and Meta-Analysis

**DOI:** 10.2196/jmir.5893

**Published:** 2016-07-21

**Authors:** Megan L Hammersley, Rachel A Jones, Anthony D Okely

**Affiliations:** ^1^ Early Start Research Institute Faculty of Social Sciences University of Wollongong Wollongong Australia

**Keywords:** overweight, obesity, child, adolescent, internet, web, online, computer, IVR, telemedicine, healthy lifestyle, dietary intake, physical activity

## Abstract

**Background:**

Effective broad-reach interventions to reduce childhood obesity are needed, but there is currently little consensus on the most effective approach. Parental involvement in interventions appears to be important. The use of eHealth modalities in interventions also seems to be promising. To our knowledge, there have been no previous reviews that have specifically investigated the effectiveness of parent-focused eHealth obesity interventions, a gap that this systematic review and meta-analysis intends to address.

**Objective:**

The objective of this study was to review the evidence for body mass index (BMI)/BMI z-score improvements in eHealth overweight and obesity randomized controlled trials for children and adolescents, where parents or carers were an agent of change.

**Methods:**

A systematic review and meta-analysis was conducted, which conforms to the Preferred Reporting Items for Systematic Reviews and Meta-analyses (PRISMA) statement. Seven databases were searched for the period January 1995 to April 2015. Primary outcome measures were BMI and/or BMI z-score at baseline and post-intervention. Secondary outcomes included diet, physical activity, and screen time. Interventions were included if they targeted parents of children and adolescents aged 0-18 years of age and used an eHealth medium such as the Internet, interactive voice response (IVR), email, social media, telemedicine, or e-learning.

**Results:**

Eight studies were included, involving 1487 parent and child or adolescent dyads. A total of 3 studies were obesity prevention trials, and 5 were obesity treatment trials. None of the studies found a statistically significant difference in BMI or BMI z-score between the intervention and control groups at post-intervention, and a meta-analysis demonstrated no significant difference in the effects of parent-focused eHealth obesity interventions compared with a control on BMI/BMI z-score (Standardized Mean Difference −0.15, 95% CI −0.45 to 0.16, Z=0.94, *P*=.35). Four of seven studies that reported on dietary outcomes demonstrated significant improvements in at least 1 dietary measurement, and 1 of 6 studies that reported on physical activity outcomes demonstrated significant improvements compared with the control. The quality of the interventions was generally not high; therefore, these results should be interpreted with caution.

**Conclusion:**

It is recommended that larger, longer duration, high-quality parent-focused eHealth studies are conducted, which transform successful components from face-to-face interventions into an eHealth format and target younger age groups in particular.

**Trial Registration:**

PROSPERO International Prospective Register of Systematic Reviews: CRD42015019837; http://www.crd.york.ac.uk/PROSPERO/display_record.asp?ID=CRD42015019837 (Archived by WebCite at http://www.webcitation.org/6ivBHvBhq)

## Introduction

The escalating global challenge of childhood obesity has been well documented, with prevalence rates climbing to approximately 23% in developed countries and 13% in developing countries [1]. Childhood is a period of time where unhealthy behaviors such as consumption of energy-dense foods and beverages, physical inactivity, and sedentary behavior are established [2]. During this time, parental influence and role modeling play a key part in the development of such behaviors [3-5]. Parental involvement in childhood obesity interventions appears to be important, given that children are highly influenced by the family unit [6,7]. Recent systematic reviews and meta-analyses have investigated the effectiveness of parent-focused childhood obesity prevention and treatment interventions, with the weight of the evidence supporting the use of parent-focused interventions. A 2012 meta-analysis of weight-related behavior change interventions for 2- to 19-year olds where parents were involved resulted in greater body mass index (BMI) reductions than interventions that had optional or no parent involvement [4]. These are similar findings to 2 meta-analyses of children aged 5-12 years [8,9], whereas another meta-analysis of 2- to 18-year olds found that interventions that targeted parents had a smaller (yet still significant) effect than those that targeted children directly [10].

The lack of studies in preschool-aged children has been highlighted [11]. Of the aforementioned 2 meta-analyses that sought to include studies, which involved children from 2 years of age, one included no studies in the preschool age group and the other included only 2 studies in this age group [4,10]. A meta-analysis of parent-focused obesity prevention and treatment interventions specifically in the early childhood (0-6 years) age group demonstrated a small, yet significant combined effect in the short term, but in the long term, the combined results were not significant [2]. When the studies were looked at individually, 5 were successful in the long term, which were all commenced at preschool age. The baseline BMI of the children appeared to be a factor, as 2 of the 3 studies that were successful at both short- and long-term follow-up included only children who were overweight or obese [2].

Effective broad-reach interventions that target childhood are required; however, currently, there is little consensus on the most effective intervention approach [11]. As mentioned, interventions that target parents are effective [2,4,8]. In addition, the use of eHealth interventions also hold promise in this area, with the use of such technology in the child and adolescent age group having increased in recent years [12]. Two previous reviews have investigated the impact of technology-based overweight and obesity interventions in childhood and adolescence with some studies reporting changes in adiposity, dietary, and/or physical activity outcomes [12,13]. However, neither of these previous reviews have specifically investigated the effect of parent involvement.

This current systematic review and meta-analysis builds on previous reviews, but differs in that it is, to our knowledge, the first to measure the efficacy of eHealth interventions in improving BMI or BMI z-score in children and adolescents where parents are an agent of change. This review is of importance in determining effective broad-reach approaches to prevent and treat childhood obesity, which in the long term could potentially alter the path of childhood obesity and reduce the progression into adult life. The review adopts a broader definition of eHealth than 1 of the previous reviews and includes interventions using the Internet, IVR (computerized voice prompts over the telephone, which participants respond to via the telephone keypad), social media (Facebook, Twitter, and so forth), mobile health (such as mobile phone apps), telemedicine (using video conferencing), email, and e-learning. The objective of this current systematic review and meta-analysis was to determine whether eHealth childhood and adolescent overweight and obesity interventions, where parents or carers are the agents of change, improved BMI and/or BMI z-scores.

## Methods

The protocol for this systematic review and meta-analysis was registered in advance with the PROSPERO international prospective register of systematic reviews (registration number CRD42015019837) and conforms to the Preferred Reporting Items for Systematic Reviews and Meta-analyses (PRISMA) statement [14].

### Eligibility Criteria

#### Type of Studies

Randomized controlled trials investigating the effect of eHealth interventions on weight of children and adolescents, where parents or carers were an agent of change, were considered for this systematic review and meta-analysis. Studies were excluded if participants had special needs or had a condition where physical activity was restricted or if they required a special diet. Studies not published in English were also excluded.

#### Type of Participants

eHealth studies targeting obesity prevention or treatment for children and adolescents aged 0-18 years, where parents or carers were agents of change, were considered. The parent or carer being an agent of change was defined as the parent or carer having an active role in the intervention and being responsible for implementing change.

#### Types of Interventions

Interventions investigating the effect of eHealth on BMI were considered for inclusion. No restrictions were placed on the type of setting, provided that the parent or carer was an agent of change.

#### Types of Outcome Measures

Primary outcome measures were BMI and/or BMI z-score at baseline and post-intervention. Secondary outcomes included body fat, waist-to-hip ratio, and improvements to dietary intake, physical activity, sedentary behavior, screen time, biomedical indicators (such as blood pressure and cholesterol), knowledge, and self-efficacy.

### Search Strategy

The electronic databases of A+ Education, CINAHL, ProQuest Central, PsycINFO, Scopus, SPORTDiscus, and Web of Science were searched with a limitation date of January 1995 to April 2015 using predetermined search terms (see Multimedia Appendix 1). Pre-1995 articles were not included as it was thought that any interventions at this early stage would be exceedingly basic. In addition, the reference lists of relevant articles were scanned.

### Study Selection

After the database searches, 1 author (MH) removed duplicates and screened the titles of the articles, and relevant articles were shortlisted. A second author (RJ) then checked the decisions made. The abstracts of the remaining articles were then screened (by MH), and a second shortlist was derived and checked by a second author (RJ). The full text of the remaining articles was retrieved and read by author one to create a final shortlist. The shortlisted articles were then viewed by the second author (RJ). Any differences were discussed, and a decision was made by consensus. Where a decision could not be reached, a third author (AO) reviewed the papers to make a final decision.

### Data Collection Process

One review author (MH) independently extracted the data from the included studies. Contact was made via email with the author of 1 paper to request additional data on BMI at a time point during the study, which was used in the meta-analysis and systematic review.

### Risk of Bias in Individual Studies

Two reviewers (AO and MH) independently assessed risk of bias using a checklist adapted from the Consolidated Standards of Reporting Trials statement (see Table 1) [15]. In line with the recommendations of the PRISMA statement, each of the items on the checklist was evaluated separately rather than an overall score being assigned. Each item was given a + or − according to whether the item was described adequately in the article (+) or not adequately described or not present (−). Any differences were discussed, and a decision was made by consensus.

**Table 1:** Risk of bias checklist.

Item	Description
A	Key baseline characteristics are presented separately for treatment groups (age, gender, and body mass index—BMI), baseline outcomes were statistically tested, and results of tests were provided
B	Randomization procedure clearly and explicitly described and adequately carried out (generation of allocation sequence, allocation of concealment, and implementation)
C	Valid measurement of BMI (at minimum, standardized method used to measure height and weight and to calculate BMI are described)
D	Dropout described and ≤20% for <6-month follow-up or ≤30% for ≥6-month follow-up
E	Blinded outcome assessment (positive when those responsible for assessing BMI were blinded to the group allocation of individual participants)
F	Intention-to-treat analysis for BMI outcome(s) (participants analyzed in group they were originally allocated to and participants were not excluded from analyses because of noncompliance to treatment or because of missing data
G	Covariates accounted for in analyses (eg, baseline score, group or cluster, and other covariates when appropriate for age or gender)
H	Summary results for each group and adjusted scores presented (adjusted difference between groups and CI)
I	Power calculation reported, and the study was adequately powered to detect hypothesized relationships

### Synthesis of Results

Extracted data were first described in a narrative manner. Studies that reported BMI or BMI z-score results as change scores or baseline and final values; standard deviation (SD), standard error (SE), or CIs; and the number of participants were included in a meta-analysis. Mean change was calculated where required, and SDs were calculated from SE or CI where SD was not reported [16]. Where the final SD value was missing, this value was imputed from baseline SD [16]. Missing SD change values were calculated using an imputed correlation coefficient [16].

Where a study had 2 eHealth intervention arms, the number of participants in the control group was divided by 2 to ensure that participants were not counted more than once in the analysis. Heterogeneity was assessed via I2 index test. The meta-analysis was conducted with reported or calculated change scores for the data collection point closest to the end of the intervention. One study was reported across 2 articles [17,18], and the time points in both these articles were used (baseline to 6 months and 6 months to 2 years—which was calculated from the available data). To enable either BMI or BMI z-score to be included in the same meta-analysis, standardized mean difference (SMD) was used. Where a study reported both BMI and BMI z-score, BMI was used. One study involved a day camp before the implementation of the eHealth intervention, and therefore, the post-camp BMI measures were used as baseline measures for the purpose of the meta-analysis to isolate this component [19]. A random effects model was applied to the analysis given the heterogeneity across the studies [16]. Analysis was conducted using Review Manager (RevMan: computer program) version 5.3; Copenhagen: The Nordic Cochrane Centre, The Cochrane Collaboration, 2014.

## Results

### Study Selection

**Figure 1:**
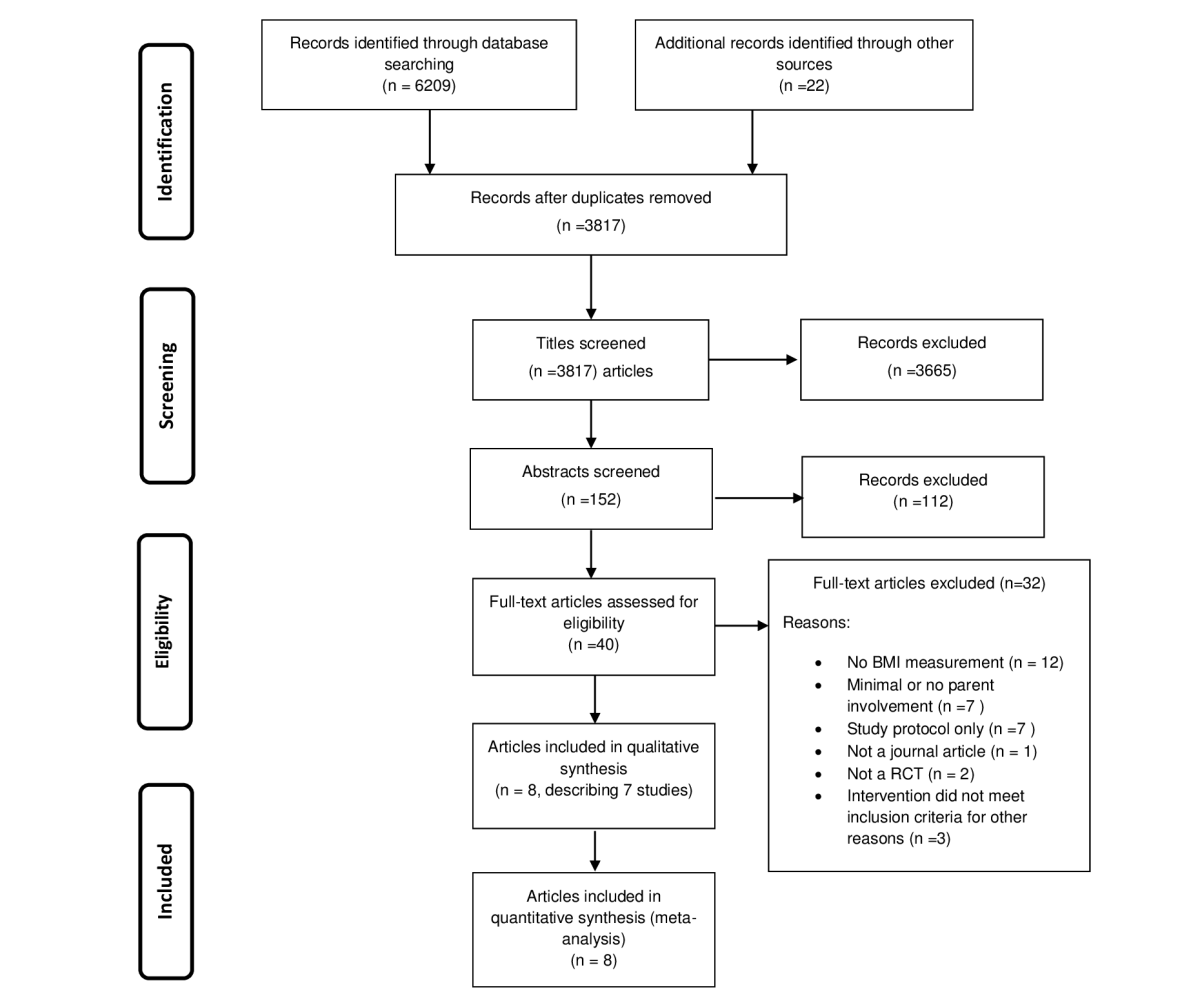
Study selection flow diagram.

From the 3817 papers that were initially identified, 8 papers describing 7 separate studies met the inclusion criteria (Figure 1).

### Description of Studies

Table 2 outlines the characteristics of the studies meeting the inclusion criteria; 7 studies were conducted in the past 10 years, and only 1 study was conducted outside the United States (in France) [22]. There were a total 1487 dyads participating in the included 8 studies (range 35-1013 dyads). A range of cultural or ethnic groups participated in studies, including African American (with 3 studies including only African American participants [17-19]), Latino [20], Chinese American (1 study included only Chinese-American participants [21]), and French [22].In total, 5 studies were overweight or obesity treatment interventions [17,18,20,23,24] and 3 studies overweight prevention interventions [19,21,22]. The gender proportions of the child or adolescent participants were 47.21% male and 52.79% female. Two of the studies included only girls [17,18]. Parent gender was reported in only 1 study [24], where 96% were female. In total, 3 studies involved children (range 7-10 years) [19,22,23], 3 studies involved adolescents (range 11-15 years) [17,18,21], and 2 studies included both children and adolescents (range 5-12 years) [20,24]. The length of the interventions ranged from 8 weeks to 2 years, with 4 studies being ≤12 weeks [19-21,24], 3 studies being ≤8 months [17,22,23] and 1 study being 2 years in duration [18]. Only 1 study collected follow-up data to assess maintenance of changes in the months after the completion of the intervention [21]. Retention rates were reported in 7 studies, and the average retention rate was 80% ± 6.3 (ranging from 70% to 93%) [17,18,20-24].

### Description of Interventions

Two of the studies had 3 study arms [20,22], and the remaining 6 studies had 2 study arms. Five studies used an Internet intervention [17-19,21,22], 2 used IVR [20,24], and 1 used telemedicine [23]. Of the Internet interventions, 1 used Internet only [21], and others used the Internet in combination with face-to-face counseling [17,18], telephone counseling, and nutrition lessons [22] or a camp [19]. The focus of behavior change differed between studies, with one focusing on diet, physical activity, and screen time [20]; 6 focusing on diet and physical activity [17-19,21-23] and 1 focusing on diet and screen time [24].

A theoretical framework underpinned 4 of the studies, 2 were underpinned by Social Cognitive Theory [19,24], 1 reported using a combination of trans-theoretical model and social cognitive theory [21], and 1 reported using social-ecological theory [20]. Studies varied in the level of detail that they provided regarding how the theory was used in the design of the intervention.

The level of parental involvement varied among studies. In 1 study, only the parents participated in the intervention (children were involved only at the data collection stages) [20]. In the remaining 7 studies, the parent and the child or adolescent both had active involvement in the intervention, either the child or adolescent participated in the eHealth activities with the parent together or there were separate components designed specifically for the parent and the child or adolescent [17-19,21-24].

Studies used differing measures of adiposity, with most using multiple measures. Six studies used BMI [17-19,21,22,24], 4 studies used BMI z-score [20,22-24], 4 studies used BMI percentile [17,18,23,24], 3 used body fat (measured by DEXA [17-19], and 1 study used waist-to-hip ratio [21]. Other measures included dietary intake (measured by food frequency questionnaire [17,18,20,24], 24-hour recall [17-19,23], or food records [21,22]) physical activity (measured by questionnaire [17-20,22] or accelerometer [19,21,23]), and screen time (measured by questionnaire [20,24]).

Three of the studies reported on the effect of higher usage of the interventions. One IVR study reported that participants who completed more calls significantly decreased their BMI z-score compared with the control group [20], whereas another IVR study reported that participants who were high IVR users demonstrated a significant reduction in BMI and BMI z-score compared with low IVR users [24]. One of the Internet studies [17] reported that change in percentage body fat was negatively correlated with use of an email facility to counselors, performance on quizzes, and use of an Internet weight monitoring function.

### Risk of Bias Within Studies

Table 3 summarizes the results of the risk of bias assessment for all included studies. Of the 8 studies, 6 reported key baseline characteristics separately for each study arm, and the results of statistical tests were provided. Seven studies reported an acceptable dropout rate (≤20% for <6-month follow-up or ≤30% for ≥6-month follow-up), and the remaining study did not report dropout rates. Six studies used intention-to-treat analysis for BMI outcomes, 7 studies accounted for covariates in the analysis; power calculations were reported and adequate in 5 articles. Only two studies described an adequate randomization procedure and/or reported summary results for each group with adjusted scores, and none of the studies described a valid, standardized method of BMI measurement.

**Table 2:** Summary of parent-focused childhood or adolescent obesity eHealth interventions.

Author, Year, Country	Participants	Intervention description	Parental involvement	Behaviors targeted	Variables measured	Key findings
Baranowski et al 2003, USA [19]	n=35, 8 years of age, girls	4-week camp with specially designed activities, followed by 8-week behavior change Internet intervention. Control girls attended camp with usual activities and a monthly Internet program with general health information and homework.	No parent involvement in camp. Intervention, and control parents had access to a website, which covered similar topics to girls’ website.	Diet (dietary fat intake, dietary fiber, water and satiety, SSB^a^), moderate to vigorous PA^b^	Demographics, body mass index (BMI), WC^c^, physical maturation, body fat (DEXA), diet (2 ×24-hour recall), PA (accelerometer and qne), preferences for PA, and SSB.	For the Internet component, no significant changes to BMI were observed. No other variables were measured at the end of the camp, so the effect of the Internet intervention on variables other than BMI could be determined.
Chen et al 2011, USA [21]	n=54, 12-15 years of age Chinese American	Behavior change Internet program with goal setting tailored to stage of change. 8 ×weekly sessions for children. Control participants accessed a general health information Internet site.	Parents received 3 Internet sessions over 8 weeks to increase knowledge and skills.	Diet (food pyramid, meal planning, portion size), PA	Parent height and weight, child BMI, waist-to-hip ratio, blood pressure, PA (accelerometer), diet (3-day food diary), PA and nutrition knowledge (qne), dietary and PA self-efficacy.	Significantly more participants in the intervention reduced their waist-to-hip ratio than the control group (effect size= −0.01, *P*=.02). There were also significant increases in PA (effect size=12.46, *P*=.01), increases to F&V^d^ intake (effect size=0.14, *P*=.001) and increased PA knowledge (effect size=0.16, *P*=.008), and nutrition knowledge (effect size=0.18, *P*=.001).
Davis et al 2013, USA [23]	n=58, 5-11 years of age, rural setting	8 × weekly telemedicine delivered psychoeducational sessions covering goal setting, diet and PA, plus 6 ×monthly sessions. Control participants visited their primary care physician to discuss set topics.	Parents met in a group separately, but at the same time as the children and covered similar content.	Nutrition (stoplight diet, portion sizes, food labels, vitamins and minerals, nutrient density), energy balance, PA, screen time, and SB^e^.	Demographics, BMI z-score, diet (24-hour recall), PA (accelerometer), child behavior checklist, behavioral pediatrics feeding assessment scale.	No statistical difference in BMI z-score between groups. There was also no significant difference between groups for kilocalories or PA.
Estabrooks et al 2009, USA [20]	n=220, 8-12 years of age	Group A: 2 × 2-hour weekly group sessions on nutrition, PA, problem-solving, and action planning delivered by dietitian. Group B: attended group sessions plus 10 interactive voice response (IVR) follow-up sessions, involving goal-setting at end of call. Both the groups received a workbook with homework on nutrition and PA topics. Control group received workbook only.	Parent was main agent of change (children participated in data collection only)	Weight, nutrition, PA, and parenting skills.	BMI z-score, PA and SB (questionnaire—qne), F&V and SSB^a^ consumption (qne), eating disorder symptoms (qne).	No significant difference in BMI z-score between groups. Significant increase in moderate-intensity physical activity in IVR group but no difference between groups. Participants completing 6-10 IVR calls significantly reduced BMI z-score compared with other groups [F(3,148)= −2.89, *P*<.01].
Paineau et al 2008, France [22]	n=1013, 7-9 years of age	All intervention families accessed a website containing information, interactive components, and other functionality. They received 30-minute dietary counseling telephone calls from a dietitian monthly for 8 months after Web-based completion of questionnaires. Children received 3 nutrition lessons at school. Children and parents received monthly newsletters. Group A: advised to reduce fat and increase complex cholesterol (CHO), Group B: advised to reduce fat and sugars and increase complex CHO. Control group received only general nutrition information at the same intervals.	Families accessed website and received mobile phone calls. Parents received monthly newsletter.	Nutrition (portions, frequency of eating, meal modification, and healthier alternatives)	Demographics, BMI, BMI z-score, body fat, WC, chest circumference, knee circumference, dietary intake (total energy, fats, sugars, complex CHO, protein) (Web-based qne and dietary records), PA (qne)	No significant difference between groups in regard to BMI or other anthropometric measures. Group A: Significantly increased complex CHO intake (mean change +10.1 (­­6.0-14.2) 95% CI, *P*<.05). Group B: Significantly reduced sugar intake (mean change −10.0 (−13.4 to −6.6) 95% CI, *P*<.01). Both groups A and B reduced total energy (mean change A −60 (−104 to −15) 95% CI, *P*<.05, B −96 (−146 to −45) 95% CI, *P*<.01) and fat intake (mean change A −8.2 (−10.6 to −5.8) 95% CI, *P*<.01, B −8.3 (−10.8 to −5.7), 95% CI, *P*<.01) compared with control group. No difference in PA between groups.
Williamson et al 2005, USA [17]	n=57, 11-15 years of age, African-American girls	Behavioral website providing nutrition information and behavior modification for 6 months. Counseling provided via email. Control group had access to general noninteractive health website. 4 face-to-face sessions over 12 weeks, focused on goal setting, behavioral contracting, monitoring of progress, and problem-solving. Control group sessions were conducted by a dietitian and included general nutrition information.	Parent and adolescent participated in the face-to-face and Internet components together	Nutrition (low energy diet, F&V, PA, food monitoring)	Demographics, BMI, BMI percentile, body fat (DEXA), eating disorders, pubertal status, dietary intake (24-hour recall and FFQ), weight loss behavior scale, child dietary self-efficacy scale, PA social support, children’s eating attitudes test, satisfaction with life scale, child depression inventory, Rosenberg self-esteem scale, Kansas family life satisfaction scale, symptom checklist-90	Participants in the intervention group lost significantly more body fat (−1.12± 0.47 standard error—SE) than the control group 0.43±0.47 SE, *P*<.05) There was a significant difference in BMI change between groups (intervention −0.19 ± 0.24 SE, <0.05, control +0.65 ± 0.23 SE, *P*<.05). Participants in the intervention group significantly reduced fat intake compared with control group (FFQ) (−145.67 ± 37.67 SE, *P*<.05)
Williamson et al 2006, USA [18]	n=57, 11-15 years of age, African-American girls	Behavioral website providing nutrition information and behavior modification over 2 years. Counseling provided via email. Control group had access to general noninteractive health website. 4 face-to-face sessions over 12 weeks, focused on goal setting, behavioral contracting, monitoring of progress, and problem-solving. Control group sessions were conducted by a dietitian and included general nutrition information.	Parent and adolescent participated in the face-to-face and Internet components together	Nutrition (low energy diet, F&V, PA, food monitoring).	Demographics, BMI, BMI percentile, body fat (DEXA), eating disorders, pubertal status, weight loss behavior scale, website use, computer opinion survey.	At 2 years, there was no significant difference in BMI, weight, or body fat. Higher BMI percentile at baseline was associated with greater reduction in BMI percentile. Higher weight loss behavior scale score at baseline was associated with greater improvement. In regard to reported consumption of fattening foods, there was a significant difference between groups (F (1,48) =2.08, *P*<.05).
Wright et al 2013, USA [24]	n=50, 9-12 years of age	Parents and children individually received 12× weekly interactive voice response (IVR) telephone counseling calls, which provided education, monitoring, and counseling on managing weight and reducing screen time. Information sent via electronic health record to the child’s pediatrician and used at visit 1 month after the intervention. Control participants attended the same pediatrician visit.	Received IVR calls independently to children.	Nutrition (energy, spotlight diet, healthy alternatives, cooking and shopping, eating out), and screen time	BMI, dietary intake (energy, fat, fruits, vegetables) (qne), TV viewing time (qne)	There was no significant difference between groups for BMI, BMI z-score, dietary intake or screen time. There was a significant difference in weight (−4.0 change, *P*=.001), BMI (−1.2 change, *P*=.01), and BMI z-score (−0.1 change, *P*=.04) between high users and low users.

^a^SSB: sugar-sweetened beverages.

^b^PA: physical activity.

^c^WC: waist circumference.

^d^F&V=fruit and vegetables.

^e^SB: sedentary behavior.

**Table 3:** Risk of bias assessment in randomized controlled trials assessing BMI outcomes of parent-focused eHealth overweight and obesity interventions.

Study	Baranowski et al 2003	Chen et al 2011	Davis et al 2013	Estabrooks et al 2009	Paineau et al 2008	Williamson et al 2005	Williamson et al 2006	Wright et al 2013
Baseline characteristics by group	**+**	−	**+**	**+**	**+**	**+**	−	**+**
Randomization described and conducted	**+**	−	−	−	−	−	−	**+**
Valid measurement of BMI	−	−	−	−	−	−	−	−
Dropout ≤20% for <6 months and ≤30% for ≥6 months	−	**+**	**+**	**+**	**+**	**+**	**+**	**+**
Blinded outcome assessment	−	−	−	−	**+**	−	−	−
Intention to treat for BMI outcomes	**+**	−	−	**+**	**+**	**+**	**+**	**+**
Covariates accounted for in analysis	**+**	−	**+**	**+**	**+**	**+**	**+**	**+**
Summary results + adjusted difference between groups + CI	**+**	**+**	−	−	−	−	−	−
Power calculation reported and power adequate	−	**+**	**+**	**+**	**+**	−	**+**	−

+ Adequately described and present.

**−** absent.

### Results of Individual Studies

#### Adiposity Outcomes

None of the included studies reported a significant difference between groups for BMI, BMI z-score, BMI percentile, or percentage body fat from baseline to the end of the eHealth intervention. One study reported a significant difference in percentage body fat between groups at 6 months (**−**1.12 ± 0.47 SE, *P*<.05) [17]; this change was not maintained at the end of the 2-year intervention [18]. One study reported a significant difference between groups for waist-to-hip ratio from baseline to the end of the intervention (effect size = **−**0.01, *P*=.02) but reported no significant difference for BMI between groups [21].

#### Dietary Outcomes

Four of the seven studies that assessed dietary intake (which were all Internet interventions) demonstrated a significant difference between groups in regard to improvement in at least 1 dietary outcome, such as fruit and vegetable intake [21], nutrition knowledge [21], total energy intake [22], fat intake [17,22] and “eating less fattening foods” [18].

#### Physical Activity Outcomes

Of the 6 studies that assessed physical activity (which was an Internet intervention), 1 study demonstrated a significant difference between groups in objectively measured physical activity and physical activity knowledge [21].

#### Screen Time Outcomes

Neither of the 2 studies that assessed screen time demonstrated a significant difference between groups for screen time [22,24].

### Synthesis of Results

**Figure 2:**
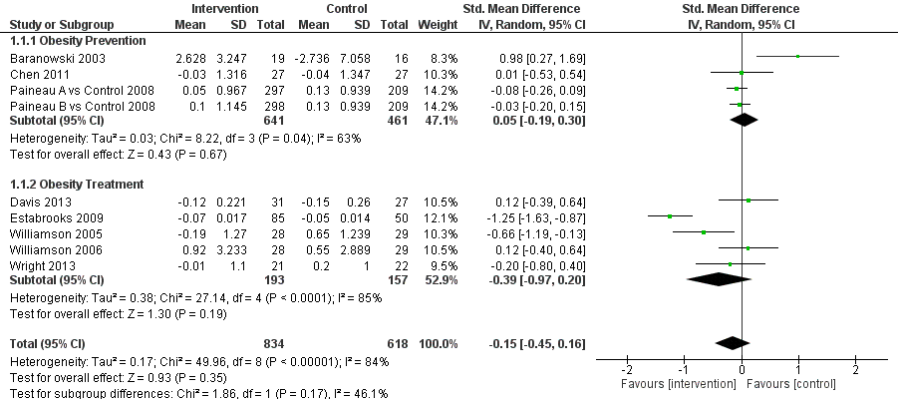
Effect of eHealth interventions on BMI or BMI z-score.

A meta-analysis was conducted on pooled data from 8 papers with a total of 9 study arms, which compared eHealth intervention groups with control groups. The meta-analysis results are displayed in Figure 2. The studies were found to be significantly heterogeneous (I2=84%, 95% CI: 71%-91%, *P*<.001). There was no significant difference in the effects of the eHealth interventions compared with the control groups on BMI/BMI z-score (SMD −0.15, 95% CI: −0.45 to 0.16, Z=0.93, *P*=.35). A sensitivity analysis was conducted by removing an outlying study [19], with heterogeneity decreasing slightly (I2=83%, 95% CI: 67%-91%, *P*<.001) and although the standardized mean difference moved toward favoring the intervention (−0.25, 95% CI −0.55 to 0.05), significance was not reached (Z=1.63, *P*=.10).

A sub-group analysis was conducted based on whether the study aim was obesity treatment or obesity prevention (refer to Figure 2). There was a larger effect for the obesity treatment studies (−0.39, 95% CI −0.97 to 0.20) compared with the obesity prevention studies (0.05, 95% CI −0.19 to 0.30), although this was not statistically significant. The obesity treatment studies appeared to have a higher level of heterogeneity (85%) than the obesity prevention studies (63%); however, given the small number of studies included, this should be interpreted with caution.

## Discussion

This meta-analysis and systematic review is, to our knowledge, the first to measure the effects of parent-focused eHealth childhood obesity interventions on BMI / BMI z-score. Overall, it was determined by meta-analysis that the included interventions did not result in significant improvements to BMI or BMI z-score compared with a control group. However, 4 of the 8 studies reported a significant improvement in at least 1 dietary or physical activity outcome measure.

The short duration of most of the studies may have meant there was insufficient time to detect changes in BMI or BMI z-score. The longest intervention demonstrated a significant improvement in body fat at the 6-month point [17], but this was not sustained at the end of the intervention at 2 years [18]. Maintenance of weight loss in the long term is indeed important but is a widespread challenge that has been well documented in both adult and child or adolescent age groups [11,25]. Previous parent-focused childhood or adolescent obesity systematic reviews and meta-analyses (which have not focused on eHealth) have highlighted the low proportion of studies, which have a follow-up period of >12 months [2-4,10,26], and 1 meta-analysis stated that there was a potential publication bias, meaning that it was suspected that some long-term follow-up studies with null results were not published [2]. Likewise, the lack of long-term follow-up studies has also been identified in childhood or adolescent obesity eHealth systematic reviews (which have not concentrated solely on parent-focused interventions), and it has been recommended that future interventions incorporate long-term follow-up in their design [12,13].

Maintaining engagement in eHealth interventions can be challenging [27]. The dropout rates in the current meta-analysis ranged from 12% to 29%. Previous childhood obesity eHealth systematic reviews have reported dropout rates up to 58% [12,13]. For participants that complete an eHealth intervention, the level of engagement as measured by usage rates can vary. Two of the studies in this review reported that higher usage rates resulted in more favorable BMI or BMI z-score outcomes [20,24], and 1 study found that body fat was negatively correlated to use of an email facility to counselors, quiz results, and weight self-monitoring [17]. Conversely, lower usage rates may therefore have impacted the effectiveness of the interventions in this review. The extent of such an effect is difficult to determine as the remaining studies did not report on the differential outcomes of high users compared with low users. It is also difficult to ascertain if those who use an intervention more do so because they are more motivated, and therefore, results of comparisons between high and low users may not necessarily be indicative of the effect of the intervention itself [20]. None of the previous eHealth or parent-focused childhood obesity systematic reviews have specifically addressed the effect of usage rates on outcomes; however, it has been demonstrated in a previous systematic review on general eHealth interventions that adhere to weight-related eHealth interventions are associated with positive outcomes [28].

Most of the studies in this current review used an eHealth modality combined with face-to-face, telephone, group sessions, workbooks, or camp activities [17-20,22-24]. Only one of the interventions used eHealth as the sole mode, and interestingly, this was the only intervention to demonstrate a significant difference between groups in an anthropometric measure at the end of the intervention, with participants in the intervention group achieving a significant reduction in waist-to-hip ratio compared with the control group [21]. In regard to the studies that used other modes in addition to the eHealth mode, in most cases, it was not possible to isolate the effects of the eHealth mode, and therefore, we were not able to determine the exact effect of the eHealth component. A previous parent-focused childhood obesity systematic review found that interventions where parents received only 1 delivery mode produced better outcomes than interventions with more than 1 mode of delivery. The authors speculated that the parents may have found the intervention to be too complex when more than 1 mode was used [2], and it is possible that this may have been the case for other studies included in this current review. Previous eHealth childhood or adolescent obesity systematic reviews have discussed isolating the effects of the eHealth intervention either only briefly or not at all. Nguyen found that of the 24 studies reviewed, only 6 used eHealth as the sole mode, and 4 of these 6 studies resulted in significant improvements in BMI, BMI z-score, or obesity-related behaviors [12].

The level of parent and child or adolescent involvement in the interventions varied, but 7 of the 8 interventions involved the children or adolescents to some degree [17-19,21-24]. Only 1 of the studies delivered the intervention solely to the parent [20]. Interestingly, this was the study that was found to have the largest effect size. However, due to the small number of studies included, it is difficult to draw any conclusions from this, particularly given that the result was not statistically significant. This is similar to findings from previous parent-focused childhood or adolescent obesity systematic reviews, most of which have found that parent-focused interventions have demonstrated better outcomes than interventions where there was either no parent involvement or it was optional [4,8,9].

Three of the studies in the current review were aimed at obesity prevention and did not have being overweight or obese as an inclusion criteria. Baseline BMI or BMI z-score was therefore lower in these studies than in studies where obesity treatment was the focus, and this may have been a factor in reporting nonsignificant findings for BMI outcomes. Understandably, a subgroup analysis indicated a larger effect for obesity treatment studies compared with obesity prevention studies, which confers with a previous parent-focused childhood obesity review, which found that interventions largely aimed at obesity prevention did not significantly reduce BMI but rather prevented increases in BMI [4]. However, both these types of studies (obesity prevention and treatment) are important.

The eHealth modality used may have been a factor in the success of an intervention; however, due to the small number of studies using particular eHealth modalities (only 1 used telemedicine and 2 used interactive voice response), a subgroup analysis was not conducted. The systematic review found that 4 of the 5 Internet interventions produced positive outcomes in waist-to-hip ratio, nutrition, or physical activity measures. Internet interventions are the widest studied of eHealth modalities and have demonstrated positive effects in other recent reviews on eHealth obesity interventions [12,29].

The effectiveness of the specific content of eHealth interventions on study outcomes has not been specifically addressed in previous eHealth childhood obesity systematic reviews. In adult populations, Internet interventions with additional components such as self-monitoring, feedback, reminders, email counseling, Web-based discussion groups, Web-based lessons, text messages, social networking, or mobile phone apps have been found to be more successful in producing weight loss outcomes. Such components were used to a small extent in the studies included in this review, including monitoring [18,21,22], email counseling [18], feedback [18], and reminders [19]. The incorporation of more of these components in future eHealth childhood obesity interventions may assist in improving outcomes.

There were no interventions targeting the early childhood age group (0-5 years) in this review, and in general childhood obesity research, there has been a lack of interventions in this age group [11]. Overall, parent-focused childhood obesity interventions have been found to be effective in this age group in the short term, particularly where only 1 mode of intervention is used [2]. It has been proposed that early childhood is the ideal life stage to intervene in the course of childhood obesity as it is a time where new healthy lifestyle practices can be introduced, rather than attempting to change well-established unhealthy practices in older age groups [5]. At this stage of life, parents are usually the main influence on the nutrition and physical activity practices of their children, and therefore, the effect of parental influence is likely to be more profound than in older age groups when outside influences become more prominent [5]. Engaging parents of young children via an eHealth modality may be an appealing format for parent-focused interventions, given that parents in developed countries with children within this age group appear to be tech savvy (as suggested by a high proportion of Internet or SMART phone use) [30-33].

There were only a small number of studies found over the 20-year period included in this meta-analysis, demonstrating that this field of study has not been well investigated, despite the dramatic advances and acceptability in technology. eHealth in childhood or adolescent obesity is only a relatively new area; a 2010 systematic review found only 21 studies, and only 11 of these were randomized controlled trials (RCTs) [12]. In this current parent-focused review, there was only 1 study found that was over 10 years old.

The quality of the interventions was generally not high, with the areas of randomization, blinded outcome assessment, valid measurement of BMI, and adjusted difference between groups either not being described or adequately carried out in a number of studies. The results should therefore be interpreted with caution due to potential bias. This is a similar finding to a previous eHealth childhood obesity review [12].

### Strengths and Limitations

The strengths of this meta-analysis and systematic review include adherence to a registered study protocol and rigorous use of the PRISMA statement. A detailed search strategy was used over several databases with a wide date range, and strict inclusion criteria were applied during the study selection process. To our knowledge, this review is the first to quantitatively measure the effects of parent-focused eHealth childhood or adolescent obesity interventions on BMI or BMI z-score. Limitations of this review include the restriction to articles published only in English, the small number of RCTs found, varying study quality, heterogeneity of the studies, inadequate power to detect an outcome in some studies due to a small number of participants, inability to isolate the effects of the eHealth component of the intervention in most studies, varying aims between studies (with some studies focusing on obesity prevention and others on obesity treatment), and all but 1 study being conducted in the United States.

In regard to the meta-analysis, as previously stated in the results, there was an outlying study that favored the control group [19]. It should be noted that this study reported a significant difference in BMI measures at baseline (with the control group having a much larger mean BMI than the intervention group), which may have influenced the results. The planned subgroup analyses comparing the type of eHealth modality used and participant age were not conducted due to the small number of studies and the wide range of ages within the individual studies making it difficult to analyze different age groups. Finally, as there were less than 10 studies in the meta-analysis, a funnel plot analysis was not conducted due to the low power of this test when there are a small number of included studies [16].

### Conclusions

This systematic review and meta-analysis found that there was no significant reduction in BMI or BMI z-score resulting from parent-focused eHealth childhood or adolescent obesity interventions compared with control. Only 1 study found a significant change in weight or adiposity measures (waist-to-hip ratio), and half of the studies demonstrated significant improvements obesity-related behaviors such as diet or physical activity compared with a control group. Only 1 study used eHealth as the sole modality, making it difficult to determine the true effect of eHealth on obesity. This review highlighted key weaknesses in the current literature: most studies were generally not of high quality, many had a short duration and lack of long-term follow-up, and many included only a small number of participants; and therefore, they may have been inadequately powered. There was an absence of studies that included children aged younger than 5 years, an age group where parental influence is probably more profound than older childhood and adolescence. It is therefore recommended that larger, high-quality studies of longer duration and longer follow-up are conducted, which transform successful components from face-to-face interventions into an eHealth format, particularly those that target younger age groups.

## Appendix

Multimedia Appendix 1 Search strategy.

## Abbreviations

BMI: body mass index
BMI z-score: body mass index z-score
CHO: carbohydrate
CI: confidence interval
FFQ: food frequency questionnaire
F&V: fruit and vegetable
IVR: interactive voice response
PA: physical activity
Qne: questionnaire
RCTs: randomized controlled trials
SSB: sugar-sweetened beverages
SD: standard deviation
SE: standard error
SMD: Standardized mean difference
WC: waist circumference
