# Supplementary material for: Parent-Focused Childhood and Adolescent Overweight and Obesity eHealth Interventions: A Systematic Review and Meta-Analysis
Source: J Med Internet Res. 2016 Jul 21;18(7):e203. doi: 10.2196/jmir.5893 (PMC4974451; doi:10.2196/jmir.5893)
Supplement: Multimedia Appendix 1 [file jmir_v18i7e203_app1.pdf]

Parent-focussed childhood and adolescent overweight and obesity interventions using eHealth: systematic review and meta-analysis

Search strategy:

1. child* OR adolescen* OR paediatric OR pediatric OR teen OR youth
2. famil* OR parent* OR Carer*
3. e-health OR eHealth OR internet OR technology OR web* OR online OR mhealth OR m-health OR mobile OR "social media" OR "social network*" OR email OR telemedicine OR e-learning OR elearning
4. *weight OR obes*OR BMI OR adipos* OR nutrition OR diet* OR activ* OR lifestyle OR "behaviour change" OR "behavior change" OR promot* OR "health behaviour" OR "health behavior"
5. RCT OR interven* OR program* OR manag* OR prevent* OR trial*
